# An Improved Two-Stage RARE Algorithm for Mixed Far-Field and Near-Field Source Localization Under Unknown Mutual Coupling with the Uniform Linear Sensor Array

**DOI:** 10.3390/s26030839

**Published:** 2026-01-27

**Authors:** Keyu Chen, Ke Deng, Jianguo Zhang

**Affiliations:** Faculty of Electronic and Information Engineering, Xi’an Jiaotong University, Xi’an 710049, China; denke@stu.xjtu.edu.cn (K.D.); jgzhang@mail.xjtu.edu.cn (J.Z.)

**Keywords:** DOA, mixed sources, mutual coupling, RARE

## Abstract

An Improved Two-Stage Rank Reduction (ITS-RARE) algorithm is proposed for the localization of mixed far-field (FF) and near-field (NF) sources under unknown mutual coupling with the uniform linear sensor array. Our algorithm includes two steps: in the first step, the eigenvectors are exploited when the rank reduction occurs at the right DOAs in our method. The eigenvectors corresponding to the smallest eigenvalues inherently represent the mutual coupling coefficient vectors. Based on it, the joint estimation of FF source DOAs and mutual coupling factors is achieved without pre-calibration. In the second step, after the DOA estimation of NF sources (NFSs), the ranges are estimated in closed form. As a result, the computational complexity is significantly reduced compared to existing methods. Furthermore, the full array aperture is preserved through the covariance matrix reconstruction (CMR) method during the FF/NF source classification. The simulation results demonstrate that the proposed algorithm is not only computationally efficient and effective in source classification but also preserves a larger effective aperture, thereby improving estimation accuracy.

## 1. Introduction

Source localization is a fundamental problem in array signal processing with wide applications. Numerous direction-of-arrival (DOA) estimation algorithms have been proposed to date. A source localization method based on Multiple Signal Classification (MUSIC) is proposed in [1]; the Estimation of Signal Parameters via Rotational Invariant Techniques (ESPRIT) is utilized for DOA estimation in [2]; and some derivative algorithms have since been proposed in [3,4].

However, the algorithms in [1,2,3,4] are only applicable to the localization of far-field (FF) sources, and the near-field (NF) ones [5] and mutual coupling [6] are not taken into account. In real scenarios, sources may be located in the NF region [5,7,8,9,10,11], and their localization requires joint estimation of both DOAs and ranges. Localization of NF sources (NFSs) has been tackled with various methods, including the two-dimensional MUSIC algorithm [7], Covariance Approximation (CA) methods [8,9], and Rank Reduction (RARE) class algorithms [10,11]. However, in scenarios like industrial fault diagnosis and environmental monitoring, FF and NF sources may coexist [12]. For such cases, a Second-Order-Statistics (SOS)-based algorithm is introduced in [13], and Fourth-Order-Cumulant (FOC)-based algorithms are proposed in [14,15], achieving better performance at the expense of higher computational complexity.

When the inter-sensor spacing is smaller than half of a wavelength, the mutual coupling effect between sensors cannot be ignored [6]. Ye et al. pioneered a method based on the intermediate subarray [16], where the DOAs can be estimated without array calibration. Subsequently, several extensions of this approach have been proposed in [17,18,19]. In [16,17,18,19], only the intermediate subarray is used; thus, the aperture is relatively small. This limitation can be overcome by the RARE-based methods in [20,21], where the full array aperture is utilized. Furthermore, novel sparse array designs are proposed in [22,23], which achieve enhanced degrees of freedom with non-uniform geometries; however, this gain often comes at the expense of either degraded estimation accuracy due to covariance matrix incompleteness or increased computational complexity associated with sparse reconstruction. Deep learning-based approaches have also been introduced in [24,25,26,27], which typically demonstrate high performance in the specific scenarios they are trained on. However, it should be noted that such approaches generally require extensive pre-training phases with considerable computational resources.

In many practical scenarios, such as those in [28], the FF and NF signals coexist and the mutual coupling is unknown. Several algorithms have been developed for this case [28,29,30]. A decoupling-based localization algorithm is proposed in [28], where the source DOA, distance, and the mutual coupling coefficients are decoupled, and three separate one-dimensional (1D) spectral peak searches must be performed. However, this algorithm is only applicable to non-circular signals. An algorithm is proposed with partially calibrated non-uniform linear array in [29], which is applied to more general cases: precise spatial geometry and non-uniform linear array. Nevertheless, preliminary partial calibration of the array is required, and to fully utilize the corrected elements, three FOC matrices are constructed. As a result, the computational complexity is relatively high. A Two-Stage RARE (TSRARE) method is proposed in [30], which enables the localization of sources by applying RARE-class algorithms in two successive stages. However, the number of eigenvectors corresponding to the NFS signal subspace is doubled, leading to a reduction in the degrees of freedom. Furthermore, in the estimation of mutual coupling coefficients, the pseudo-inverse of matrices is required, and after the DOA estimation of NFSs, the spectral peak search is required for the estimation of ranges; thus, the computational complexity is relatively high. Moreover, in [6,7,8,9,10,11,12,13,14,15,16,17,18,19,20,21,22,23,24,25,26,27,28,29,30], the mutual coupling coefficients are not jointly estimated with the DOAs of FFSs, and additional computation is required.

In this paper, an Improved Two-Stage RARE (ITS-RARE) algorithm is proposed for uniform linear sensor arrays. The key contributions are outlined below:

1. Calibration-free joint estimation of FFSs and mutual coupling coefficients:

Differently to [30], the eigenvectors are exploited when the rank reduction occurs at the right DOAs in our method. The eigenvectors corresponding to the smallest eigenvalues inherently represent the mutual coupling coefficient vectors. Thus, the computational burden is decreased significantly in the estimation of mutual coupling coefficients compared with conventional methods like [16,30]. Based on it, the joint estimation of FFS DOAs and mutual coupling factors is achieved without pre-calibration.

2. High-aperture, low-complexity localization of NFSs:

During the source classification, FFS information is effectively eliminated, avoiding the expansion of the NFS source signal subspace. Thus, more degrees of freedom are preserved, thereby improving array aperture utilization. Moreover, the ranges of NFSs are estimated in closed form rather than spectral peak searches, significantly reducing the computational complexity.

This paper is organized as follows: the model for mixed FFSs and NFSs under unknown mutual coupling is presented in Section 2; the ITS-RARE algorithm is described in Section 3; the performance is shown and compared with existing algorithms through simulations in Section 4; and the paper is concluded in Section 5.

$(\cdot {)}^{*},(\cdot {)}^{T},(\cdot {)}^{H}$ represent the conjugate, transpose, and conjugate transpose operations, while $(\cdot {)}^{-1}$, $(\cdot {)}^{\#}$ denote the inverse and pseudo-inverse operations, respectively.

## 2. Signal Model

A uniform linear array (ULA) composed of *N =* 2*M* + 1 elements is considered, arranged symmetrically about the origin and the distance between sensors is *d*. K independent narrowband signals impinge on the receiving array, including *K*_1_ FFSs and *K*_2_ NFSs.

(1) $$x_{m}(t)=\sum_{k=1}^{K}s_{k}(t)e^{j\tau_{mk}}+n_{m}(t)$$

where $s_{k}(t)$ denotes the *k*-th signal, $n_{m}\left(t\right)$ represents the noise term, and $\tau_{mk}$ represents the phase shift experienced by the *k*-th signal as it propagates from the phase reference point to the *m*-th array element [1]:

(2) $$\tau_{mk}=\frac{2\pi }{\lambda }\left(\sqrt{r_{k}^{2}+{\left(md\right)}^{2}-2r_{k}\mathrm{md}\sin\theta_{k}}-r_{k}\right)$$

where $\theta_{k}$ is the DOA of the *k*-th signal, $r_{k}$ denotes the distance from the *k*-th signal to the reference sensor, and λ represents the wavelength.

When the *k*-th signal is the FFS, the $\tau_{mk}$ can be expressed as

(3) $$\tau_{mk}\approx -\frac{2\pi md}{\lambda }\sin\theta_{k}.$$

In practical scenarios, signal sources often reside within the NF region, and the expression in (3) is no longer applicable. In this case, $\tau_{mk}$ can be approximated by

(4) $$\tau_{mk}\approx -\frac{2\pi }{\lambda }mdsin\theta_{k}+\frac{\pi {\left(md\right)}^{2}}{\lambda r_{k}}\cos^{2}\theta_{k}=m\omega_{k}+m^{2}\Phi_{k}$$

where

(5) $$\omega_{k}=-\frac{2\pi d}{\lambda }\sin\theta_{k}$$

(6) $$\Phi_{k}=\frac{\pi d^{2}}{\lambda r_{k}}\cos^{2}\theta_{k}.$$

In real scenarios, mutual coupling inevitably exists between array elements [6]. In lots of studies [6,28,29,30], the distance between adjacent elements is fixed at λ/4, and the mutual coupling must be taken into account [6]. Just like in [6], a symmetric Toeplitz matrix **C** with dimensions *N* × *N* is employed to characterize the mutual coupling among array elements. And we have

(7) $$c={\left[\begin{array}{c} c_{0},c_{1},c_{2},\ldots ,c_{P} \end{array}\right]}^{T}$$

(8) $$C=\mathrm{Toeplitz}\{c\}=\left[\begin{array}{ccccccc} 1 & c_{1} & \cdots & c_{P} & 0 & \cdots & 0\\ c_{1} & 1 & c_{1} & \vdots & \ddots & \ddots & \vdots \\ \vdots & c_{1} & 1 & \ddots & \vdots & \ddots & 0\\ c_{P} & \vdots & \ddots & \ddots & \ddots & \vdots & c_{P}\\ 0 & \ddots & \vdots & \ddots & 1 & c_{1} & \vdots \\ \vdots & \ddots & \ddots & \vdots & \vdots & 1 & c_{1}\\ 0 & \cdots & 0 & c_{P} & \cdots & c_{1} & 1 \end{array}\right]$$

where *P* is the maximum number of adjacent array elements involved in mutual coupling and Toeplitz{**c**} signifies the construction of a symmetric Toeplitz matrix **C** from the vector. NF sources are typically located close to the array, resulting in stronger yet more localized mutual coupling effects; thus, *P* is generally set to 2 or 3 [28,30]. In this paper, *P* is set to 3, which provides enhanced adaptability to diverse scenarios and array configurations.

When mutual coupling is taken into account, the received signal **x**(*t*) can be expressed as

(9) $$\begin{array}{l} x(t)={\left[x_{-M}(t),\ldots ,x_{0}(t),\ldots ,x_{M}(t)\right]}^{T}\\ =\sum_{i=1}^{K_{1}}Ca(\theta_{i},\infty )s_{i}(t)+\sum_{j=K_{1}+1}^{K}Ca(\theta_{j},r_{j})s_{j}(t)+n(t)\\ =CA_{FF}s_{FF}(t)+CA_{NF}s_{NF}(t)+n(t)\\ =CAs(t)+n(t). \end{array}$$

$A=[A_{FF},A_{NF}]$ is the steering matrix for the mixed signals, where $s(t)={\left[s_{FF}^{T}(t),s_{NF}^{T}(t)\right]}^{T}$, $s_{FF}(t)={\left[s_{1}(t),\ldots ,s_{K_{1}}(t)\right]}^{T}$, $s_{NF}(t)={\left[s_{K_{1}+1}(t),\ldots ,s_{K}(t)\right]}^{T}$ and $n(t)={\left[n_{-M}(t),\ldots ,n_{M}\right]}^{T}$ is the noise vector. We have

(10) $$A_{FF}=\left[\begin{array}{c} a(\theta_{1},\infty ),\ldots ,a(\theta_{k},\infty ),\ldots ,a(\theta_{K_{1}},\infty ) \end{array}\right]$$

(11) $$A_{NF}=\left[\begin{array}{c} a(\theta_{K_{1}+1},r_{K_{1}+1}),\ldots ,a(\theta_{K_{1}+k},r_{K_{1}+k}),\ldots ,a(\theta_{K},r_{K}) \end{array}\right]$$

(12) $$a(\theta_{k},r_{k})={\left[e^{j\left(-\omega_{k}M+\Phi_{k}M^{2}\right)},\ldots ,1,\ldots ,e^{j\left(\omega_{k}M+\Phi_{k}M^{2}\right)}\right]}^{T}.$$

In the following sections, the steering vector $a(\theta_{k},\infty )$ of the FFSs will be simplified to $a(\theta_{k})$.

To analyze the problem rigorously, the following assumptions are presented:

(1) The incoming signals are statistically independent, have zero mean, and exhibit stationarity.
(2) The noise is modeled as additive white Gaussian with zero mean and is statistically uncorrelated with the source signals.
(3) The inter-element spacing is set to d≤λ/4 to ensure no phase ambiguity ($\Delta \Phi_{\max}=\pi /2<\pi$) [30]. Another feasible approach to resolve the phase ambiguity is to employ a data model fitting method [31]: when phase ambiguity occurs, two candidate solutions are obtained, and the correct one is selected based on which better fits the observed data model.

## 3. ITS-RARE Algorithm

Our algorithm consists of two steps: firstly, under unknown mutual coupling, both the incident angles of FFSs and sensor coupling parameters are jointly estimated by an improved RARE algorithm, achieving a higher effective aperture than the methods in [16,17,18,19] and lower computational complexity compared to those in [20,21]. Secondly, after the compensation of mutual coupling matrix, the covariance matrix of the FFSs is reconstructed and then subtracted from the total received covariance matrix. This approach effectively eliminates the information of FFSs and preserves the degrees of freedom, in contrast to the differential subtraction methods in [13,30]. After the DOA estimation of NFSs with the RARE algorithm, the corresponding ranges are obtained in closed form rather than spectral peak search.

### 3.1. Localization of FFSs in the Presence of Unknown Mutual Coupling

With the aid of (9) and the previously stated assumptions, the received signal’s covariance matrix can be expressed as

(13) $$\begin{array}{l} R_{x}=E\{x(t)x^{H}(t)\}=CAR_{s}A^{H}C^{H}+\sigma^{2}I_{N}=R_{FF}+R_{NF}+\sigma^{2}I_{N}\\ \;\;\;\;\;=\underset{R_{FF}}{\underbrace{CA_{FF}S_{FF}A_{FF}^{H}C^{H}}}+\underset{R_{NF}}{\underbrace{CA_{NF}S_{NF}A_{NF}^{H}C^{H}}}+\sigma^{2}I_{N} \end{array}$$

where $S_{FF}=E\{s_{FF}(t)s_{FF}^{H}(t)\},S_{NF}=E\{s_{NF}(t)s_{NF}^{H}(t)\}.$ $R_{s}=blkdiag\{S_{FF},S_{NF}\}$ takes a block diagonal structure.

Performing the eigenvalue decomposition on $R_{x}$, we have

(14) $$R_{x}=U_{s}\Lambda_{s}U_{s}^{H}+\sigma_{n}^{2}U_{n}U_{n}^{H}$$

where $\Lambda_{S}$ is a diagonal matrix composed of *K* dominant eigenvalues; $U_{n}$ denotes the noise subspace of $R_{x}$ with dimensions *N* × (*N − K*); and $U_{s}$ represents the signal subspace of $R_{x}$ with dimensions *N* × *K*.

Obviously, the signal subspace, which is spanned by $U_{s}$, is equivalently represented by $CA_{FF}$ and remains orthogonal to the noise subspace spanned by $U_{n}$. This orthogonality leads to the following result:

(15) $${\left|U_{n}^{H}Ca(\theta_{k})\right|}^{2}=0,\;k=1,\ldots ,K_{1}.$$

Ca(θ) can be rewritten as

(16) $$Ca(\theta )=T_{a}(\theta )c,$$

wherein, $T_{a}(\theta )=T_{1}(\theta )+T_{2}(\theta )$ is the sum of two matrices:

(17) $${\left[T_{1}(\theta )\right]}_{p,q}=\left\{\begin{array}{c} \begin{array}{ll} {\left[a(\theta )\right]}_{p+q-1} & \mathrm{for}\;p+q\leq N+1\\ 0 & \mathrm{otherwise} \end{array} \end{array}\right.$$

(18) $${\left[T_{2}(\theta )\right]}_{p,q}=\left\{\begin{array}{c} \begin{array}{ll} {\left[a(\theta )\right]}_{p-q+1} & \mathrm{for}\;p\geq q\geq 2\\ 0 & \mathrm{otherwise} \end{array} \end{array}\right..$$

Combining (15) and (16), we have

(19) $$\begin{array}{l} a^{H}(\theta )C^{H}(c)U_{n}U_{n}^{H}C(c)a(\theta )\\ =c^{H}T_{a}^{H}(\theta )U_{n}U_{n}^{H}T_{a}(\theta )c\\ =c^{H}G(\theta )c \end{array}$$

where G(θ) is a conjugate symmetric non-negative definite matrix with dimensions (*P* + 1) × (*P* + 1) defined as

(20) $$G(\theta )=T_{a}^{H}(\theta )U_{n}U_{n}^{H}T_{a}(\theta ).$$

Since c≠0, we have [30]:

(21) $$\left\{\begin{array}{l} c^{H}G(\theta )c=0\;\mathrm{only}\;\mathrm{when}\;\mathrm{rank}[G(\theta )]<P+1\\ c^{H}G(\theta )c=0\;\mathrm{only}\;\mathrm{when}\;\theta =\theta_{k}. \end{array}\right.$$

Thus, when $\theta =\theta_{k}$, det[G(θ)]=0. And the determinant of G(θ) can be obtained by

(22) $$\det[G(\theta )]=\lambda_{1}\lambda_{2}\ldots \lambda_{P+1},$$

where $\lambda_{1}\lambda_{2}\ldots \lambda_{P+1}$ are the eigenvalues of G(θ), sorted from the largest to the smallest. Thus, when $\theta =\theta_{k}$, the smallest eigenvalue $\lambda_{P+1}=0$. In brief, the following spectral peak search can be constructed:

(23) $$P_{FF}(\theta )=\frac{1}{\lambda_{P+1}}.$$

Since a total of $K_{1}$ FFSs exist, the $K_{1}$ smallest eigenvalues are equal to 0. Thus, the $K_{1}$ largest values of $P_{FF}(\theta )$ correspond to the DOAs of the FFSs.

### 3.2. Estimation of Mutual Coupling Coefficients

In the estimation of mutual coupling coefficients, we have the following theorem:

**Theorem** **1.**  *The non-zero vector of mutual coupling coefficients corresponds to the eigenvector linked to the minimum eigenvalue of $G(\theta_{k})$*.

**Proof.**  See Appendix A. □

In Section 3.1, when the DOAs of FFSs are obtained, the eigenvector linked to the smallest eigenvalue is jointly obtained. Based on Theorem 1, the mutual coupling coefficients can be calculated in the DOA estimation of FFSs. The $K_{1}$ largest values of $P_{FF}(\theta )$ are found at $\theta =\theta_{k}$; thus, when $P_{FF}(\theta )$ takes the $K_{1}$ largest values, the non-zero mutual coupling coefficient vector is the eigenvector linked to the smallest eigenvalue of $G(\theta_{k})$. To achieve better results, an average operation is performed:

(24) $$c=\frac{1}{K_{1}}\sum_{k=1}^{K_{1}}c_{k},$$

wherein $c_{k}$ is the mutual coupling coefficient vector at $\theta =\theta_{k}$. The method above simplifies the complexity of the algorithm.

After the estimation of **c**, the mutual coupling matrix can be reconstructed as **C** = Toeplitz{**c**}, and the mutual coupling can be then compensated with

(25) $$R=C^{-1}(R_{x}-\sigma^{2}I_{N})C^{-H}=A_{FF}S_{FF}A_{FF}^{H}+A_{NF}S_{NF}A_{NF}^{H},$$

where $\sigma^{2}$ is the average of the *N* − *K* smallest eigenvalues in $R_{x}$ and denotes the noise power [32]. Through (25), the mutual coupling is compensated, which helps improve the accuracy of subsequent source separation and NFS localization.

### 3.3. Classification of FFSs and NFSs

If the FFS components are not removed, they will be jointly estimated with the NFSs. Then, the number of resolvable NFSs is reduced and the effective aperture is decreased, thereby degrading the estimation accuracy. Therefore, the FFSs must be eliminated before the localization of NFSs, and the details are as follows.

As established in Section 2, due to the mutual statistical independence among sources, the covariance matrix can be decomposed into non-interfering components corresponding to FF and NF sources:

(26) $$R=\underset{\mathrm{FF}\;\mathrm{component}}{\underbrace{A_{FF}S_{FF}A_{FF}^{H}}}+\underset{\mathrm{NF}\;\mathrm{component}}{\underbrace{A_{NF}S_{NF}A_{NF}^{H}}},$$

After the estimation of $\theta_{1},\ldots ,\theta_{K_{1}}$, their steering vector $a(\theta_{1}),\ldots ,a(\theta_{K_{1}})$ can be reconstructed, and then their power can be calculated by [32]

(27) $$\begin{array}{ll} \sigma_{k}^{2} & ={\left\{{\left[A^{\#}a(\theta_{k})\right]}^{H}R_{s}^{\#}[A^{\#}a(\theta_{k})]\right\}}^{-1}\\ & ={\left\{a^{H}(\theta_{k}){\left[U_{s}(\Lambda_{s}-\sigma^{2}I_{K\times K})U_{s}^{H}\right]}^{\#}a(\theta_{k})\right\}}^{-1}. \end{array}$$

Following the estimation of power $\sigma_{k}^{2}$ of each FFS, the covariance matrix can be reconstructed as

(28) $$R_{FF}=A_{FF}\mathrm{diag}(\sigma_{1}^{2},\sigma_{2}^{2},\ldots ,\sigma_{K_{1}}^{2})A_{FF}^{H}.$$

Then, the covariance matrix of the NFSs can be calculated by

(29) $$R_{NF}=R-R_{FF}.$$

Since the reconstruction of $R_{FF}$ relies solely on FF parameters and the independence assumption ensures no cross-terms between FFSs and NFSs, this subtraction effectively removes the contribution of FF sources while preserving the full spatial structure of $R_{NF}$.

Crucially, when the mutual coupling coefficients and DOAs of FFS are estimated with high accuracy, the reconstructed $R_{FF}$ accurately matches its true value, ensuring that $R_{NF}$ contains only the NF signal information. As a result, the number of eigenvectors spanning the NF signal subspace equals the number of NF sources, enabling the use of the entire array aperture for NF localization.

### 3.4. DOA Estimation of NFSs

Due to the symmetry of the uniform linear array with respect to the central sensor [30], the steering vector of the NF source $a(\theta_{k},r_{k})$ can be expressed as

(30) $$a(\theta_{k},r_{k})=V(\theta_{k})g(\theta_{k},r_{k})$$

wherein $V(\theta_{k})$ is a matrix with dimensions (2*M* + 1) × (*M* + 1) containing only DOA information and $g(\theta_{k},r_{k})$ is a column vector with M+1 elements:

(31) $$V(\theta_{k})=\left[\begin{array}{ccccc} \exp(-jM\omega_{k}) & \ldots & 0 & \ldots & 0\\ \vdots & \ddots & \vdots & \ldots & \vdots \\ 0 & \ldots & \exp(-jm\omega_{k}) & \ldots & \vdots \\ \vdots & \ldots & \vdots & \ddots & \vdots \\ 0 & \ldots & 0 & 0 & 1\\ \vdots & \ldots & \vdots & \ddots & \vdots \\ 0 & \ldots & \exp(jm\omega_{k}) & \ldots & \vdots \\ \vdots & \ddots & \vdots & \ldots & \vdots \\ \exp(jM\omega_{k}) & \ldots & 0 & \ldots & 0 \end{array}\right]$$

(32) $$g(\theta_{k},r_{k})={\left[e^{j\Phi_{k}M^{2}},\ldots ,e^{j\Phi_{k}m^{2}},\ldots ,1\right]}^{T}.$$

Performing the eigenvalue decomposition on $R_{NF}$, we have

(33) $$R_{NF}=E_{s}\Delta_{s}E_{s}^{H}+E_{n}\Delta_{n}E_{n}^{H}$$

where $E_{n}$ spans the noise subspace and $E_{s}$ spans the signal subspace. $\Delta_{s}$ contains the *K*_2_ dominant eigenvalues along its diagonal, and $\Delta_{n}$ is a diagonal matrix formed by the *N* − *K*_2_ zero-valued eigenvalues.

Obviously, matrix $E_{s}$ and $A_{NF}$ both span the signal subspace, which remains orthogonal to the noise subspace spanned by $E_{n}$. Then, we have

(34) $$\begin{array}{l} a^{H}(\theta_{k},r_{k})E_{n}E_{n}^{H}a(\theta_{k},r_{k})\\ =g^{H}(\theta_{k},r_{k})V^{H}(\theta_{k})E_{n}E_{n}^{H}V(\theta_{k})g(\theta_{k},r_{k})\\ =g^{H}(\theta_{k},r_{k})Q(\theta_{k})g(\theta_{k},r_{k})\\ =0 \end{array}$$

where $Q(\theta_{k})$ is defined as

(35) $$Q(\theta_{k})=V^{H}(\theta_{k})E_{n}E_{n}^{H}V(\theta_{k}).$$

Similar to Section 3.1, since g ≠ 0 and **Q** is a symmetric positive semidefinite matrix, Q will be rank-deficient only when θ =${\;\theta }_{k}$. When Q is rank-deficient, its smallest eigenvalue $\sigma_{\min}=\;0$. Thus, a spectral peak search can be performed on the reciprocal of the smallest eigenvalue of Q, and the $K_{2}$ largest values of P(θ) will correspond to $\theta_{k},k\;=\;K_{1}\;+\;1\;,\ldots ,\;K$:

(36) $$P(\theta )=\frac{1}{\sigma_{min}}$$

### 3.5. Range Estimation of NFSs

After the DOA estimation of NFSs, the next step is to estimate their ranges. According to [33], the subspace spanned by $g\left(\theta_{k},r_{k}\right)$ can also be spanned by the eigenvector $v_{k}$ associated with the smallest eigenvalue of $Q\left(\theta_{k}\right)$. Due to the properties of eigenvectors, $v_{k}$ and $g\left(\theta_{k},r_{k}\right)$ span the same subspace, and they differ by a complex scalar factor. Since the last entry of $g\left(\theta_{k},r_{k}\right)$ is 1, this discrepancy can be eliminated by dividing vector $v_{k}$ by its last element, such that the last element equals 1 (i.e., its phase is set to 0):

(37) $${\hat{v}}_{k}=\frac{v_{k}}{e^{j∠(v_{k,N})}}.$$

Then, its phase can be calculated as

(38) $$\begin{array}{ll} {\hat{a}}_{k} & =\mathrm{angle}({\hat{v}}_{k})\\ & ={\left[N^{2}\Phi_{k},{\left(N-1\right)}^{2}\Phi_{k},\ldots ,\Phi_{k},0\right]}^{T}. \end{array}$$

A matrix, $T_{1}$, is defined as

(39) $$T_{1}={\left[\begin{array}{ccccc} 1 & 1 & 1 & 1 & 1\\ N^{2} & {\left(N-1\right)}^{2} & \cdots & 1 & 0 \end{array}\right]}^{T}.$$

The objective at this point is to find $\left[\begin{array}{c} \delta_{k}\\ {\hat{\Phi }}_{k} \end{array}\right]$, which makes ${∥T_{1}\left[\begin{array}{c} \delta_{k}\\ {\hat{\Phi }}_{k} \end{array}\right]-{\hat{a}}_{k}∥}_{F}^{2}$ minimum. Herein, $\delta_{k}$ is the error parameter, and ${\hat{\Phi }}_{k}$ is the target value. Since $T_{1}$ is of full column rank, a left pseudo-inverse of matrix $T_{1}$ exists. Thus, $\left[\begin{array}{c} \delta_{k}\\ {\hat{\Phi }}_{k} \end{array}\right]$ can be obtained by

(40) $$\left[\begin{array}{c} \delta_{k}\\ {\hat{\Phi }}_{k} \end{array}\right]=T_{1}^{\#}{\hat{a}}_{k}={\left(T_{1}^{T}T_{1}\right)}^{-1}T_{1}^{T}{\hat{a}}_{k}.$$

Then, the range ${\hat{r}}_{k}$ can be obtained:

(41) $${\hat{r}}_{k}=\frac{\pi d^{2}}{\lambda {\hat{\Phi }}_{k}}\cos^{2}{\hat{\theta }}_{k}.$$

In practical scenarios, the covariance matrix is typically estimated by the average of sample covariance matrices over *L* snapshots:

(42) $${\hat{R}}_{x}=\frac{1}{L}\sum_{l=1}^{L}\{x(t)x^{H}(t)\}.$$

Poposed ITS-RARE method can be summarized as Algorithm 1:
**Algorithm 1.** ITS-RARE1: Estimated the covariance matrix ${\hat{R}}_{x}$ is by (42).2: Perform the eigenvalue decomposition on ${\hat{R}}_{x}$ to get ${\hat{U}}_{n}$.3: Obtained the DOAs of FFSs are by (23).4: Estimated the non-zero mutual coupling coefficient vector is by (24).5: Compensated the impact of mutual coupling is effectively by (25).6: Reconstructed the covariance matrix of the FFSs is by (28).7: Classify the sources and get the covariance matrix of the NFSs by (29).8: Perform the eigenvalue decomposition on ${\hat{R}}_{NF}$ to get ${\hat{E}}_{n}$.9: Estimate the DOAs of NFSs by (36).10: Estimate the ranges of NFSs by (41).

### 3.6. Computational Complexity

For the proposed method and methods in [16,30], the computational complexity of different components is analyzed as follows:

- In the DOA estimation stage of FFSs, the computational complexity primarily arises from the construction of G(θ) and spectral peak search in proposed method and method in [30]. The complexity of constructing G(θ) is $O(N^{2})$ and for an angular search step size of degrees $\theta_{\Delta }$, a total of 180/$\theta_{\Delta }$ spectral searches are required; thus, the total computational complexity of this stage is $O(\frac{180N^{2}}{\theta_{\Delta }})$ (since G(θ) is a square matrix with dimensions (*P +* 1) × (*P* + 1) and *P* is typically 2 or 3, the computational complexity of calculating its determinant and performing eigenvalue decomposition is negligible). As for the method in [16], similarly, its computational complexity originates from matrix construction and spectral searches. Due to an iterative procedure that performs the spectral search twice, the computational complexity is $O(\frac{180{\left(N-2P+2\right)}^{2}}{\theta_{\Delta }}+\frac{180N^{2}}{\theta_{\Delta }})$.
- In the estimation stage of mutual coupling coefficients, in the proposed method, the mutual coupling coefficients are estimated simultaneously with the DOAs of FFSs; thus, this stage incurs no additional overhead. However, in [16,30], the construction and pseudo-inverse of a matrix with dimensions K (2M + 2P − 1) × P is needed. The computational cost is KNP for matrix construction and $KNP^{2}$ for pseudo-inverse computation. Therefore, the total computational complexity is $O(KNP^{2})$.
- In the DOA estimation stage of NFSs, the same as the DOA estimation of FFSs, the matrix Q(θ) is constructed and a spectral peak search is performed on it. Thus, the computational complexity in the proposed method and the method in [16] for this stage is $O(\frac{180N^{2}}{\theta_{\Delta }})$.
- In the range estimation stage of NFSs, in the proposed method, firstly, the phase of ${\hat{v}}_{k}$ is extracted. Subsequently, matrix **T** is constructed (since **T** is fixed, it just needs to be constructed once and the computational complexity is negligible), and its pseudo-inverse is computed. The computational cost of phase exaction and pseudo-inverse is N and 2N, respectively; thus, the total computational complexity for all NFSs is $O(K_{2}N)$. As reported in [30], after the DOA estimation of $K_{2}$ NFSs, spectral peak searches are conducted by substituting the estimated DOAs into the expression $\max{\left[a^{H}(\theta_{k})E_{n}E_{n}^{H}a(\theta_{k})\right]}^{-1}$, where $E_{n}E_{n}^{H}$ is precomputed. The computational complexity of evaluating $a^{H}(\theta_{k})E_{n}E_{n}^{H}a(\theta_{k})$ at each range point is $N^{2}$. Given that a spectral search needs to be performed within the NF region, which spans approximately $0.62\sqrt{\frac{D^{3}}{\lambda }}\sim \frac{2D^{2}}{\lambda }$, for a range search step size of $r_{\Delta }$, the computational complexity of a spectral peak search is roughly $\frac{\frac{2D^{2}}{\lambda }-0.62\sqrt{\frac{D^{3}}{\lambda }}}{r_{\Delta }}N^{2}$. Therefore, the total computational complexity for the range estimation of $K_{2}$ NFSs is $O(K_{2}\frac{\frac{2D^{2}}{\lambda }-0.62\sqrt{\frac{D^{3}}{\lambda }}}{r_{\Delta }}N^{2})$.
- In the stage of source classification, in the proposed method, steering vectors $a(\theta_{1}),\ldots ,a(\theta_{K_{1}})$ are reconstructed through the estimated DOAs, and the computational complexity is $K_{1}N$. Since the noise has been eliminated in the preceding steps, power estimation only requires subsequent operations, with a computational complexity of $N^{3}$ for the pseudo-inverse operation and $N^{2}$ for the matrix multiplication operation; thus, the total computational complexity is $O(N^{3})$. As reported in [30], first, matrix **R** needs to be multiplied twice with an exchange matrix (i.e., a matrix with ones on the anti-diagonal and zeros elsewhere). In practice, this operation only requires reordering the elements of **R**, resulting in a computational complexity of $N^{2}$. The subsequent matrix subtraction and conjugate operations both have a computational complexity of $N^{2}$; thus, the total computational complexity is $O(N^{2})$.
  The computational complexity is shown in Table 1.

**Figure 1:**
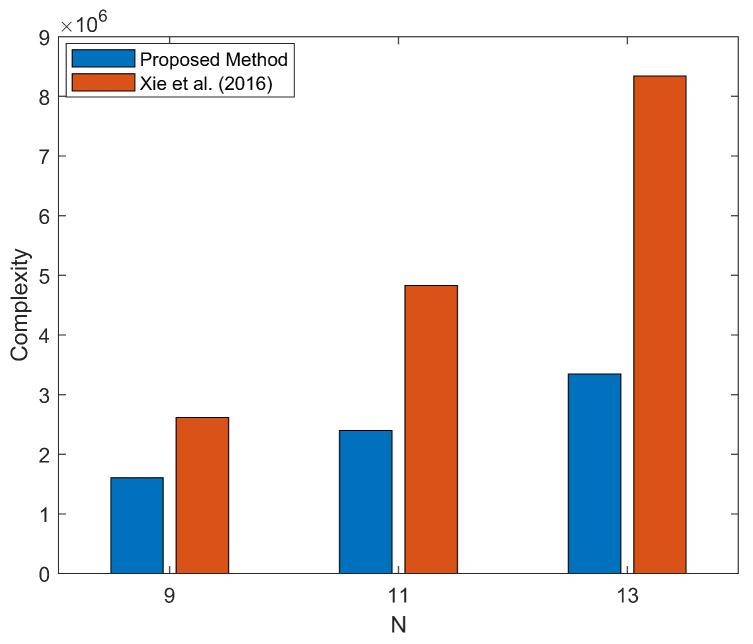
Complexity comparison of proposed method and Xie et al. (2016) [30].

In Figure 1, the computational complexity of the proposed method is compared with that in [30], considering four incident signals—two FFSs and two NFSs with *P* = 3. The angle search step $\theta_{\Delta }$ and range search step $r_{\Delta }$ are set to 0.1° and 0.001, respectively. The computational complexity of the proposed method is significantly lower than that in [30], and the gap between two methods widens as *N* increases.

Table 2 presents the total running time of the proposed method and the method in [30] for different numbers of array elements *N* under identical hardware conditions (Intel i7-13620H, 16 GB RAM), where simulations span an SNR range from −6 dB to 20 dB in 4 dB steps, with 200 snapshots and 200 Monte Carlo trials conducted at each SNR level.

The results show that the computational time of our method is shorter than that of the method in [30], and the time difference between them increases with the number of array elements.

## 4. Simulation Results

The performance of our algorithm is examined through detailed simulations and comparative studies in this section. The uniform linear array is used with 11 elements (i.e., 5 elements on each side of the phase reference), and the element spacing $d\;=\;\frac{\lambda }{4}$. The signals are mutually independent narrowband waveforms, and the SNR, root mean square error (RMSE), and root mean square (RMS) are defined as follows:

(43) $$\mathrm{SNR}=10\mathrm{lg}\frac{\sigma_{k}^{2}}{\sigma^{2}}$$

(44) $$\mathrm{RMSE}=\sqrt{\frac{1}{GT}\sum_{g=1}^{G}\sum_{t=1}^{T}{\left(y_{gt}^{*}-y_{g}\right)}^{2}}$$

(45) $$\mathrm{RMS}=\sqrt{\frac{1}{GT}\sum_{g=1}^{G}\sum_{t=1}^{T}p_{gt}^{*2}}$$

wherein $\sigma_{k}^{2}$ denotes the power of the *k*-th signal, and $\sigma^{2}$ is the noise power. $y_{gt}^{*}$ is the *t*-th estimated DOA or range for the *g*-th signal; $y_{g}$ is the actual DOA or range for the *g*-th signal; and $p_{gt}^{*}$ is the *t*-th estimated power for the *g*-th signal. To enhance the robustness and reliability of the simulation results, all simulations in this paper are conducted with 200 Monte Carlo trials across different scenarios and condition. All RMSE and RMS values are averaged over all sources, and DOA-related RMSE values are expressed in degrees.

In this simulation, the DOAs and ranges of sources are listed in Table 3. To ensure the reliability and robustness of the experiments, in each Monte Carlo trial, the DOAs are perturbed by up to ±3° from their nominal values, and the ranges of the NFSs are shifted by up to ±0.3 wavelengths from their original values:

As mutual coupling strength generally diminishes with increasing inter-element spacing, the nominal mutual coupling vector is modeled as c = [1, 0.3489 + 0.4487 i, 0.2587 − 0.3657 i, 0.1587 + 0.0657 i]. To evaluate robustness, the off-diagonal coefficients are perturbed in each Monte Carlo trial by up to ±20% in magnitude and ±20° in phase, simulating realistic variations due to manufacturing tolerances or environmental effects.

### 4.1. Performance of FFS DOA Estimation Under Mutual Coupling

From Section 3.1, it is known that when $\theta =\;\theta_{k}$, $k=\;1,\;2,\;\ldots ,{\;K}_{1},$ $G(\theta_{k})=0$. In this case, the condition number $\frac{\lambda_{1}}{\lambda_{P+1}}$ should be the maximum, and the minimum eigenvalue $\lambda_{P+1\;}=\;0$. Based on (21), the following three spectral functions can be constructed:

1. $P_{1}(\theta )=\frac{1}{\det[G(\theta )]}$;
2. $P_{2}(\theta )=\frac{\lambda_{1}}{\lambda_{P+1}}$;
3. $P_{3}(\theta )=\frac{1}{\lambda_{P+1}}$.

**Figure 2:**
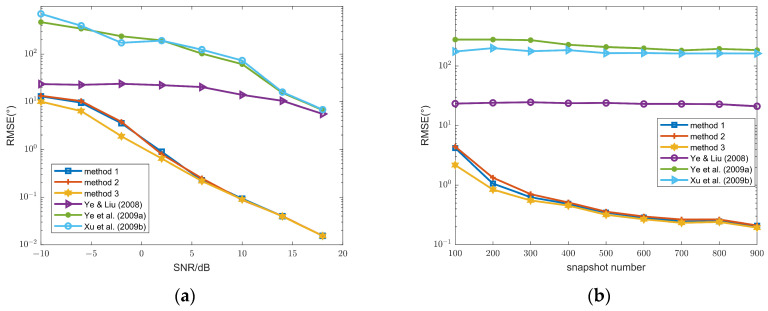
(**a**) DOA RMSEs of the FFSs versus SNR, snapshot number = 100; (**b**) DOA RMSEs of the FFSs versus snapshot number, SNR = 10 dB; methods 1–3 and methods in Ye & Liu (2008) [16], Ye et al. (2009a) [17] and Xu et al. (2009b) [18].

To compare the performance of three different methods, RMSEs versus SNR and snapshot number are compared in Figure 2. The performance of these three methods is superior to those in [16,17,18]. Moreover, methods 1–3 exhibit an SNR threshold of 5 dB; when the SNR is greater than or equal to 5 dB, their estimation accuracy is sufficiently high to meet practical requirements. In contrast, the methods in [16,17,18] fail to achieve this SNR threshold under the same conditions. The reason is that only a central subarray with *N* − 2(*P* + 1) sensors is utilized in [16,17,18], whereas the full array is exploited in these methods.

From (a), for SNR values below 6 dB, the RMSE of method 3 is significantly lower than that of the other two methods. From (b), method 3 also outperforms the other two approaches when the snapshot varies from 100 to 900. Moreover, our algorithm adopts Method 3, whereas Method 1 is employed in [30]; consequently, the proposed ITS-RARE algorithm achieves superior performance compared to the method in [30].

**Figure 3:**
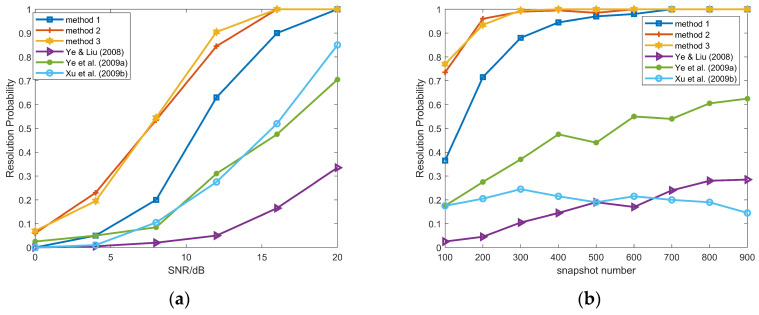
(**a**) Resolution probability of the FFSs versus SNR, snapshot number = 100; (**b**) resolution probability of the FFSs versus snapshot number, SNR = 10 dB; methods 1–3 and methods in Ye & Liu (2008) [16], Ye et al. (2009a) [17] and Xu et al. (2009b) [18].

To further compare the performance of these methods, the resolution capability is evaluated in Figure 3, with the angular separation between two FFSs fixed at 5°. A trial is considered a successful resolution if both FFSs are correctly estimated and the angle estimation error for each source is less than half of their angular separation (i.e., <2.5°). Method 3 (used in the proposed method) and method 2 achieve higher resolution than method 1 (used in [30]), and all three methods outperform those in [16,17,18].

### 4.2. Performance of Source Classification

**Figure 4:**
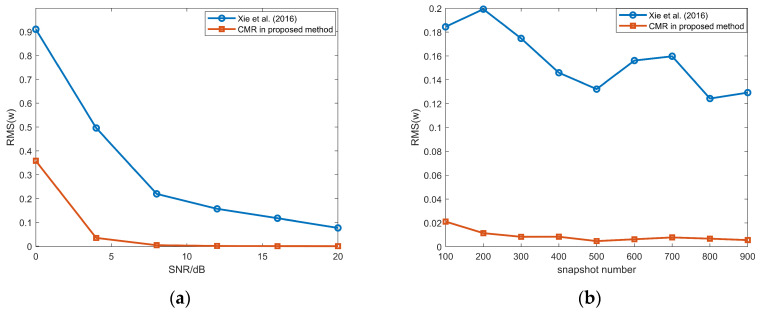
(**a**) Power RMSs of the FFSs versus SNR after different source classification methods, snapshot number = 100; (**b**) powers RMSs of the FFSs versus snapshot number after different source classification methods, SNR = 10 dB; proposed method and method in Xie et al. (2016) [30].

Figure 4 shows the RMSs of FFS powers. When the SNR varies from 0 dB to 20 dB and the snapshot number varies from 100 to 900, after CMR classification method, the RMSs of FFS powers are much lower than those after separation by the differential method in [30]. This demonstrates that our CMR method can more effectively remove the information of FFSs.

### 4.3. Performance of NFS Estimation

**Figure 5:**
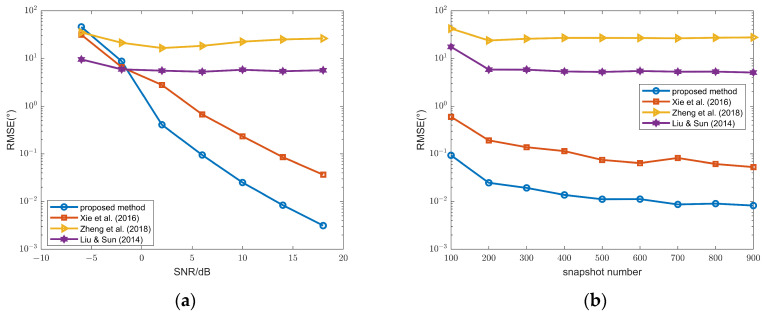
(**a**) DOA RMSEs of the NFSs versus SNR, snapshot number = 100; (**b**) DOA RMSEs of the NFSs DOAs versus snapshot number, SNR = 10 dB; proposed method and methods in Xie et al. (2016) [30], Zheng et al. (2018) [14], and Liu & Sun (2014) [13].

Figure 5 illustrates the RMSEs of DOA estimates for the NFSs, which shows that our method and the method in [30] outperform the methods in [13,14]. The reason is the lack of mutual coupling compensation in [13,14]. Figure (a) illustrates that the performance of the proposed method gradually surpasses that of the method in [30] as the SNR increases beyond −2 dB. From (b), our method outperforms the one in [30] obviously when the snapshot number varies from 100 to 900.

**Figure 6:**
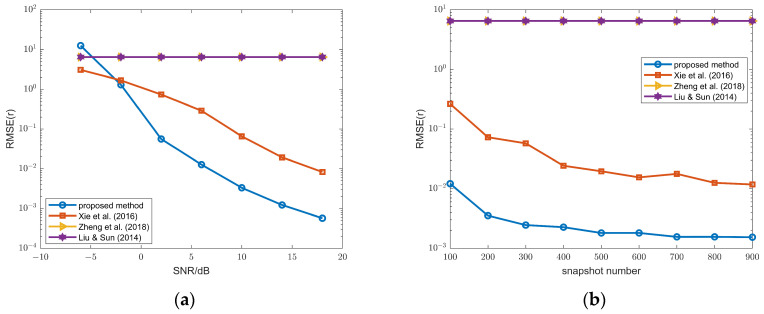
(**a**) Range RMSEs of the NFSs versus SNR, snapshot number = 100; (**b**) range RMSEs of the NFSs versus snapshot number, SNR = 10 dB; proposed method and methods in Xie et al. (2016) [30], Zheng et al. (2018) [14], and Liu & Sun (2014) [13].

The range RMSEs of the NFSs are shown in Figure 6. Similar to the DOA estimates of NFSs, our method achieves superior performance than the methods in [13,14,30].

**Figure 7:**
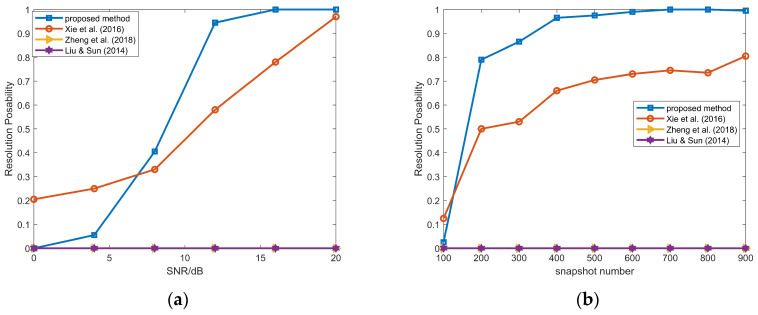
(**a**) Resolution probability of the NFSs versus SNR, snapshot number = 100; (**b**) resolution probability of the NFSs versus snapshot number, SNR = 10 dB; proposed method and methods in Xie et al. (2016) [30], Zheng et al. (2018) [14], and Liu & Sun (2014) [13].

The resolution capability of NFSs is also evaluated in Figure 7, with the angular separation between two NFSs fixed at 5°. From (a), the proposed method outperforms tge method in [30] when the SNR is greater than 5; from (b), the proposed method outperforms the method in [30] when the snapshot number is more than 100.

### 4.4. Extended Simulation—More Array Elements and Signal Sources

To enhance the reliability of the simulation results, the aforementioned scenario is extended to a scenario with *N* = 13 array elements, 2 FFSs and 3 NFSs, with the source distribution shown in Table 4.

**Figure 8:**
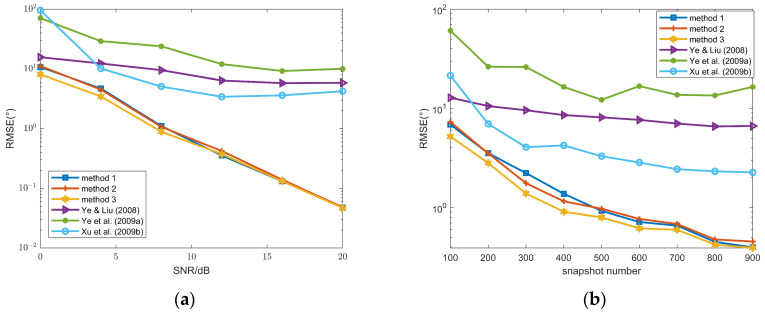
(**a**) DOA RMSEs of FFSs versus SNR, snapshot number = 100; (**b**) DOA RMSEs of FFSs versus snapshot number, SNR = 5 dB; methods 1–3 and methods in Ye & Liu (2008) [16], Ye et al. (2009a) [17] and Xu et al. (2009b) [18].

The DOA RMSEs of FFSs with are illustrated in Figure 8. Similar to Figure 3, method 3 still outperforms method 1 and method 2, and all three methods are superior to those in [16,17,18].

**Figure 9:**
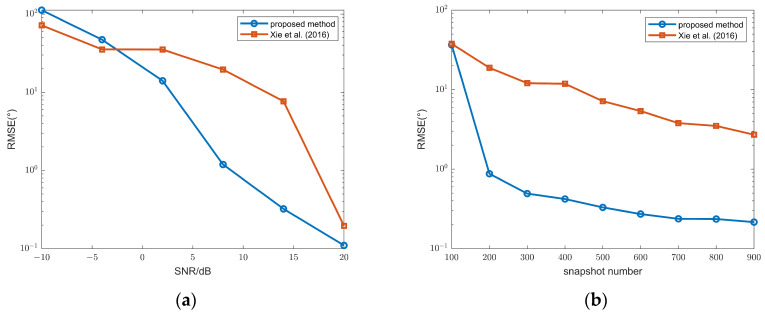
(**a**) DOA RMSEs of NFSs versus SNR, snapshot number = 200; (**b**) DOA RMSEs of NFSs versus snapshot number, SNR = 10 dB; proposed method and method in Xie et al. (2016) [30].

The DOA RMSEs of NFSs with are illustrated in Figure 9. In this scenario, the proposed method still significantly outperforms the method in [30], benefiting from the high-precision source classification approach.

These simulation results indicate that our method not only exhibits superior performance but also possesses broad applicability.

### 4.5. Performance of NFS Estimation with No Mutual Coupling

**Figure 10:**
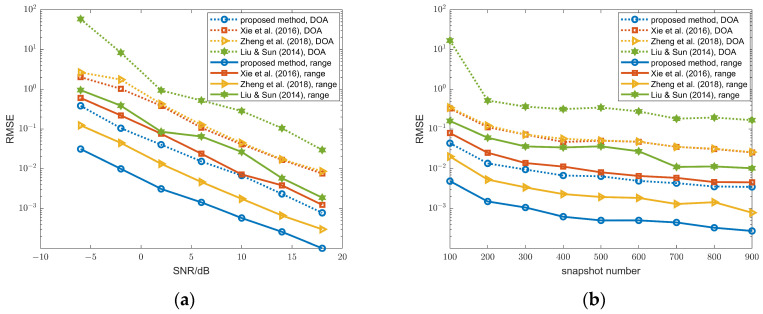
(**a**) Range and DOA RMSEs of NFSs versus SNR, snapshot number = 100; (**b**) range and DOA RMSEs of NFSs versus snapshot number, SNR = 10 dB; proposed method and methods in Xie et al. (2016) [30], Zheng et al. (2018) [14], and Liu & Sun (2014) [13].

The range and DOA RMSEs of NFSs with no mutual coupling are illustrated in Figure 10, and the parameters of signal sources are identical to those in Table 3. The DOA and range estimates of NFSs in our method are still superior, indicating that our approach provides superior NF source localization performance. The reason is that a high-precision CMR source classification method is used, effectively removing the information of FFSs and retaining information of NFSs.

### 4.6. Performance of Mutual Coupling Estimation

**Figure 11:**
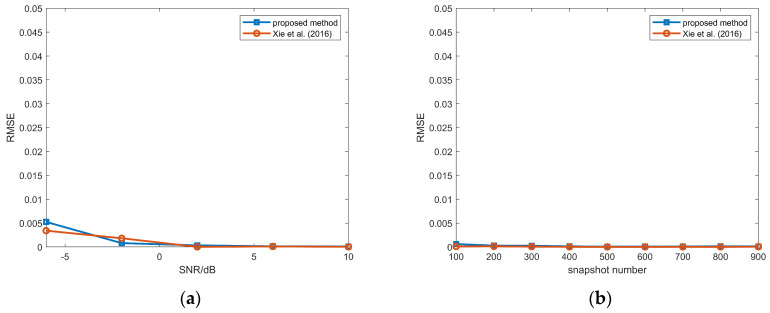
(**a**) RMSEs of mutual coupling coefficient versus SNR, snapshot number = 100; (**b**) RMSEs of mutual coupling coefficient estimation versus snapshot number, SNR = 5 dB; proposed method and method in Xie et al. (2016) [30].

The RMSEs of mutual coupling coefficients are illustrated in Figure 11. In addition to avoiding extra computations during the estimation process, high estimation accuracy for mutual coupling is still maintained by our method.

## 5. Conclusions

Focusing on the localization of mixed FFSs and NFSs with unknown mutual coupling, a novel ITS-RARE algorithm is presented. The key contributions are summarized as follows:

Calibration-free joint estimation of FFS DOAs and mutual coupling coefficients: The joint estimation of FFS DOAs and mutual coupling coefficients is achieved in our method, without the prior array calibration. Simulation results show that higher accuracy in the DOA estimation of FFSs is achieved with reduced computational complexity, while performance is maintained in the estimation of mutual coupling coefficients comparable to existing methods. High-resolution NFS localization with enhanced aperture utilization and low computational load: Through the reconstruction of the covariance matrix and the classification of FFSs and NFSs through the CMR method, the interference of FFSs is effectively suppressed while NFS information is preserved in our method. Furthermore, the dimension of NFS subspace is retained in the proposed method, thereby enhancing the utilization of array aperture and the accuracy of NFS localization. After the DOA estimation, the ranges of NFSs are estimated in closed form, avoiding the spectral peak searches. Simulation results show that the proposed method achieves superior NF localization accuracy.

## Figures and Tables

**Table 1:** Computational complexity comparison.

Steps	ITS-RARE	TSRARE in [30]	Method in [16]
DOA estimation of FFSs	$O(\frac{180N^{2}}{\theta_{\Delta }})$	$O(\frac{180N^{2}}{\theta_{\Delta }})$	$O(\frac{180{\left(N-2P+2\right)}^{2}}{\theta_{\Delta }}+\frac{180N^{2}}{\theta_{\Delta }})$
Estimation of mutual coupling coefficients	*0*	$O(KNP^{2})$	$O(KNP^{2})$
DOA estimation of NFSs	$O(\frac{180N^{2}}{\theta_{\Delta }})$	$O(\frac{180N^{2}}{\theta_{\Delta }})$	*/*
Range estimation of NFSs	$O(K_{2}N)$	$O(K_{2}\frac{\frac{2D^{2}}{\lambda }-0.62\sqrt{\frac{D^{3}}{\lambda }}}{r_{\Delta }}N^{2})$	/
Source classification	$O(N^{3})$	$O(N^{2})$	

**Table 2:** Actual running time (in seconds).

Number of Array Elements	Proposed Method	Method in [30]
*N* = 9	57.052	62.858
*N* = 11	62.515	86.402
*N* = 13	73.852	110.952

**Table 3:** DOAs and ranges of sources.

Signal	DOA	Range
${\mathrm{FF}}_{1}$	−20° ± 3°	/
${\mathrm{FF}}_{2}$	−60° ± 3°	/
${NF}_{1}$	20° ± 3°	2λ ± 0.3λ
${NF}_{2}$	0° ± 3°	3λ ± 0.3λ

**Table 4:** DOAs and ranges of sources – more sources and array elements.

Signal	DOA	Range
${\mathrm{FF}}_{1}$	$-20^{\circ }\;\pm \;3^{\circ }$	/
${\mathrm{FF}}_{2}$	$-60^{\circ }\;\pm \;3^{\circ }$	/
${NF}_{1}$	$20^{\circ }\;\pm \;3^{\circ }$	2λ ± 0.3λ
${NF}_{2}$	${-30}^{\circ }\;\pm \;3^{\circ }$	3λ ± 0.3λ
${NF}_{3}$	${-45}^{\circ }\;\pm {\;3}^{\circ }$	4λ ± 0.3λ

## Data Availability

The data presented in this study are available within the article.

## Appendix A

To prove Theorem 1 rigorously, we formulate an optimization problem that characterizes the mutual coupling coefficient vector **c**. Since the quadratic form $c^{H}G(\theta_{k})c$ equals zero for the true coupling coefficients and $G(\theta_{k})$ is positive semidefinite, this suggests that **c** minimizes this quadratic form.

Formulate the constrained optimization problem as

(A1) $$\underset{c}{\min}c^{H}G(\theta_{k})c,∥c{∥}^{2}=c^{H}c=\alpha .$$

where α is the norm of c and is independent of θ. This constraint ensures a non-trivial solution can be found. Define the Lagrangian function as

(A2) $$L(c,\mu )=c^{H}G(\theta_{k})c-\mu (c^{H}c-\alpha )$$

where μ is the Lagrange multiplier. To find the stationary points, the derivative is taken with respect to $c^{H}$:

(A3) $$\frac{\partial L}{\partial c^{H}}=G(\theta_{k})c-\mu c=0$$

Then, we can get

(A4) $$G(\theta_{k})c=\mu c.$$

From (A4), μ is the eigenvalue of $G(\theta_{k})$ and **c** is the eigenvector corresponding to μ. Then, we have

(A5) $$c^{H}G(\theta_{k})c=c^{H}\mu c=\mu \alpha .$$

To minimize $c^{H}G(\theta_{k})c$, μ should be the minimum; i.e., μ is the minimum eigenvalue and **c** is the eigenvector corresponding to the minimum eigenvalue.
